# Myofibroblasts and their relationship with oral squamous cell carcinoma

**DOI:** 10.5935/1808-8694.20130019

**Published:** 2015-10-14

**Authors:** Priscilla Suassuna Carneiro Lúcio, Alessandro Leite Cavalcanti, Pollianna Muniz Alves, Gustavo Pina Godoy, Cassiano Francisco Weege Nonaka

**Affiliations:** MSc student (Dentistry Graduate Program - State University of Paraíba - UEPB - Campina Grande - PB - Brazil); PhD (Professor - Dentistry Graduate Program - State University of Paraíba - UEPB - Campina Grande - PB - Brazil); PhD (Professor - Dentistry Graduate Program - State University of Paraíba - UEPB - Campina Grande - PB - Brazil)

**Keywords:** carcinoma, squamous cell, immunohistochemistry, myofibroblasts

## Abstract

Myofibroblasts are hybrid-phenotype differentiated cells in between fibroblasts and smooth muscle cells. Due to their contractile features and ability to synthesize extracellular matrix components, cytokines, proteases, and proangiogenic factors, myofibroblasts have been implicated in the pathogenesis of fibrocontractive diseases and in the progression of many tumors, including oral squamous cell carcinoma (SCC).

**Objective:**

To perform a literature review on the origin of myofibroblasts, their main morpho-physiological and immunohistochemical aspects, and to discuss the correlations with oral SCC.

**Method:**

A search was made on the PUBMED database to select the main papers in the literature in English related to the subject, published between January 1991 and December 2011.

**Conclusion:**

Myofibroblasts are an important component of the stroma of oral SCCs, although they are not present in all tumors. Abundant presence of myofibroblasts may be associated with local disease recurrence and decreased patient survival. However, given the relatively limited number of studies on the subject, further research is needed to clarify the molecular mechanisms by which myofibroblasts influence the biological behavior of oral SCC.

## INTRODUCTION

Neoplasms are made of tumor cells wrapped in a stroma constituted by blood vessels, fibroblasts, extracellular matrix (ECM), inflammatory cells and, occasionally, myofibroblasts^1^, ^2^, ^3^, ^4^. Even though the stroma has been considered for a long time as a support tissue to neoplastic cells, recent evidence indicates that it has a role in promoting malignant phenotypes^2^^,^^4^^,^^5^. Stromal alterations are frequently seen in many types of neoplasms, including oral squamous cell carcinoma (SCC)^6^.

Myofibroblasts were originally thought to be variants of fibroblasts present in the granulation response of the wound healing process, due to their contractile activity. Today, myofibroblasts are considered to be hybrid phenotype cells with fibroblast and smooth muscle tissue characteristics, capable of expressing α-smooth muscle actin (α-SMA), and characterized by intense synthesis of ECM proteins, growth factors - such as hepatocyte growth factor (HGF), platelet-derived growth factor (PDGF), and keratinocyte growth factor (KGF) - and proteases^1^^,^^2^^,^^7^^,^^8^.

Recent studies have shown that poorer prognosis in some tumor types is correlated with the presence of myofibroblasts in a neoplastic stroma^2^^,^^5^^,^^9^. An accepted explanation for such event is the ability of the products synthesized by these cells to modulate a number of biological events associated with malignancy, such as growth, differentiation, adhesion, migration, and tumor cell invasion^1^^,^^4^^,^^6^^,^^10^^,^^11^.

Additionally, studies have indicated that myofibroblasts may be potential targets in the treatment of malignant disease^12^, ^13^, ^14^. Among the main advantages related to targeting myofibroblasts in cancer treatment is the increased genetic stability of these cells and their wide applicability in different tumor types^14^. Strategies suggested to prevent strong interaction between myofibroblasts and malignant neoplastic cells include the inhibition of the signaling pathways involved in myofibroblast recruitment and differentiation and direct eradication of this cell type^12^^,^^13^.

### Objective

This literature review aimed to look into the origin of myofibroblasts, their main morpho-physiological and immunohistochemical aspects, and to discuss the correlation between myofibroblasts and oral SCC.

## METHOD

A search was made on database PUBMED using the following keywords based on the Medical Subject Headings (MeSH): *myofibroblasts, oral squamous cell carcinoma* and *immunohistochemistry.* Papers meeting the following criteria were included: a) data reported on myofibroblasts, their morpho-physiological and immunohistochemical aspects, and/or correlations between myofibroblasts and development/progression of oral SCC; b) study included *in vivo* or *in vitro* model;c) papers published between January 1991 and December 2011. Forty-three papers met the criteria and were selected for analysis.

## LITERATURE REVIEW

### Origin

The replacement or expansion of myofibroblasts, during homeostasis or disease, was originally believed to occur solely at the cost of the cell populations residing in the tissues, fibroblasts more particularly^15^, ^16^, ^17^. However, depending on the nature of the tissue and other characteristics of the microenvironment, several cell types may act as myofibroblast precursors, such as smooth muscle cells, pericytes^18^^,^^19^, endothelial cells^15^, pancreatic and hepatic stellate cells^4^^,^^15^, adipocytes, and myoepithelial cells^4^. Studies have also shown other potential sources of myofibroblasts, including mesenchymal stem cells residing in the tissues, bone marrow-derived fibrocytes, and epithelial-mesenchymal transition^15^, ^16^, ^17^^,^^19^^,^^20^.

The differentiation of fibroblasts residing in myofibroblasts is induced by paracrine signaling triggered by tissue repair or inflammation. Members of the transforming growth factor β (TGF-β) family - PDGF, insulin-like growth factor II (IGF-II), and interleukin-4 (IL-4) - seem to be the main factor involved in the differentiation process of fibroblasts into myofibroblasts^1^^,^^2^^,^^12^. Special attention is given to TGF-β1, a multifunctional peptide which regulates various cell activities, including growth and differentiation, and ECM macromolecule expression and metabolization^21^^,^^22^. TGF-β1 triggers the production of fibronectin by fibroblasts, which by its turn triggers the synthesis of α-SMA that is incorporated to stress fibers. Then new proteins are synthesized to allow molecular adhesion between α-SMA myofilaments, cytoplasmic membrane, and fibronectin, thus characterizing fully-differentiated myofibroblasts^8^^,^^20^.

### Morpho-physiological and immunohistochemical aspects

Myofibroblasts are cells that physiologically secrete high levels of cytokines, growth factors, chemokines, hormones, inflammatory mediators, adhesion proteins, and ECM proteins^2^^,^^7^. Myofibroblasts are present in small quantities in most organs, in particular in those in which mechanical strength is required: the oral cavity, the skin, the gastrointestinal tract, the uterus, the testes, and the heart^20^^,^^21^.

Morphologically, myofibroblasts are elongated or stellate cells with pale eosinophilic cytoplasm and an even, centrally located nucleus, with minor indentations and small nucleoli^12^. Ultrastructurally, myofibroblasts are characterized by the presence of a contractile apparatus organized in the form of bundles of peripheral α-SMA myofilaments, analogue to the stress fibers seen on in-vitro cultured fibroblasts. These myofilaments are connected to a specialized adhesion complex located on the cellular surface, called fibronexus^16^^,^^17^^,^^20^. In these structures myofilaments connect to integrins, which then bind to fibronectin in the ECM. In functional terms, this contractile apparatus provides for a mechanotransduction system to transmit the forces generated by the stress fibers to the ECM^17^^,^^23^^,^^24^.

Similarly to active fibroblasts, the myofibroblast cell has well-developed rough endoplasmic reticulum and Golgi apparatus, as a result of intense protein synthesis and secretion, particularly collagen^17^^,^^20^. As in smooth muscle cells, the presence of gap junctions allows for adhesion and electrochemical communication between myofibroblasts, although they do not have an external lamina^12^^,^^17^^,^^20^^,^^23^.

Marker α-SMA has been described as the most relevant molecule in identifying differentiated myofibroblasts^4^^,^^15^, though it cannot tell them from smooth muscle cells^15^^,^^17^. Myofibroblasts are usually negative for antigens present in smooth muscle cells, such as heavy myosin chains, caldesmon, and desmin^16^^,^^18^^,^^20^. However, studies have shown that myofibroblasts, particularly in more advanced stages of differentiation, may express antigens seen in smooth muscle cells^23^^,^^25^, revealing that the pattern of expression of these proteins may vary depending on the site and nature of the pathological process. Typically, myofibroblasts are negative for cytokeratins (epithelial cell markers), CD68 (monocyte/ macrophage marker), CD31 (endothelial cell marker), and CD34^4^^,^^12^^,^^26^.

In the 1990s, smoothelin - a protein associated with the contractile apparatus - was considered a marker for smooth muscle cell late differentiation, whose expression was not seen in myofibroblasts^27^^,^^28^. Further research found that lung fibroblasts treated with TGF-β1 could express smoothelin^29^. More recently, the 4Ig isoform of protein palladin in stress fibers was proposed as a new marker for myofibroblast differentiation^30^. However, a Western blotting protocol using antibodies against various forms of palladin protein found expression of this isoform in smooth muscle cells^31^. According to Hinz et al.^15^, no cytoskeletal proteins have been found to differentiate myofibroblasts from smooth muscle cells yet.

### Myofibroblasts and oral squamous cell carcinoma

Myofibroblasts have been identified as important components in the stroma of various solid malignant tumors, such as breast carcinoma, hepatocellular carcinoma, and melanoma^2^^,^^4^^,^^11^. Nonetheless, few papers have looked into the presence of myofibroblasts in oral SCC and the possible impact myofibroblasts have on the biological behavior of these neoplasms. Most of these papers were published only within the last five years^3^^,^^5^^,^^13^^,^^32^, ^33^, ^34^, ^35^, ^36^.

Lewis et al.^6^ studied *in vitro* cell lines of oral SCC and normal primary oral fibroblasts and verified that neoplastic cells - through TGF-β1 secretion - can induce fibroblast differentiation into myofibroblasts. The authors also observed that myofibroblasts secreted greater quantities of HGF when compared to primary fibroblasts, and that HGF promoted oral cancer cell invasion into a Matrigel matrix. According to the authors, these findings imply the existence of a double paracrine mechanism between oral cancer cells and myofibroblasts.

Kellermann et al.^8^ used an experimental model similar to the one employed by Lewis et al.^6^, and found supporting evidence for the important role of oral cancer cells in the differentiation process of normal primary fibroblasts into myofibroblasts through TGF-β1 secretion. Additionally, the authors noticed that the treatment of oral cancer cell lines with myofibroblast conditioned medium led to significant increases in tumor cell proliferation rates. According to Kellermann et al.^8^, the results reported in their study revealed the existence of mutual paracrine effects between oral cancer cells and normal oral fibroblasts, characterized by the triggering of transdifferentiation of the latter cell types into myofibroblasts and the modulation of malignant neoplastic cell proliferation.

Studies done using animal models also revealed evidence suggesting malignant epithelial cells have a special role in the appearance of myofibroblasts in oral cancer patients. Vered et al.^37^ carried out an experimental study on carcinogenesis with 4-Nitroquinoline 1-oxide (4NQO) in the tongue mucosa of Wistar rats and observed that in areas of normal, hyperkeratotic/ hyperplastic, and dysplastic epithelium, myofibroblasts were scarce or completely absent in the underlying connective tissue. However, in areas of superficial basal cell or invasive carcinoma, a significant increase on the number of myofibroblasts was seen, with close proximity between these cell types and malignant neoplastic cells. According to the authors, these findings suggested the occurrence of significant epithelial-mesenchymal interactions simultaneously to malignant transformation or possible transdifferentiation of malignant epithelial cells into myofibroblasts during oral carcinogenesis.

In addition to the findings reported previously, Vered et al.^35^ observed, in an experimental study using 4NQO in the lingual mucosa of rats, that the oral carcinomas of desalivated rats had significantly fewer myofibroblasts when compared to carcinomas of rats without salivary flow disorder. According to the authors, the presence of a significant number of myofibroblasts in oral SCC appears not to depend only on factors secreted by malignant epithelial neoplastic cells, but also on the participation of chemical mediators found in saliva, particularly TGF-β.

Kellermann et al.^3^ used immunohistochemistry to look into the presence of myofibroblasts in tongue SCC, oral epithelial dysplasia, and normal mucosa specimens. Unlike in normal oral mucosa and oral epithelial dysplasia specimens, myofibroblasts were observed only in the stroma of carcinomas, particularly in the tumor invasion front. Additionally, lower survival rates were seen in patients whose tumors had abundant amounts of myofibroblasts in the tumor invasion front. Similar results were obtained in a study done by Kawashiri et al.^33^ on SCCs of various regions of the oral cavity. According to Kellermann et al.^3^, analyzing the number of myofibroblasts in tongue SCC may be useful in determining patient prognosis.

Vered et al.^35^ used immunohistochemistry to analyze the presence of myofibroblasts in tongue SCC specimens and observed higher tumor incidence with greater scores of myofibroblasts in patients aged under 60 years. The authors also saw that the 5-year disease-free survival rates of patients whose tumors had more myofibroblasts were lower (37.6% *vs*. 82.3%). Additionally, the percentage of survivors five years after diagnosis was significantly lower in the group with tumors with more myofibroblasts (61.2%) when compared to the group with fewer myofibroblasts (90.6%). Multivariate regression analysis revealed that abundance of myofibroblasts in the tumor stroma had an independent adverse impact on local recurrence rates. According to Vered et al.^35^, abundance of myofibroblasts in tongue SCC stroma increases the risk of local recurrence, particularly in patients under 60 years of age.

## DISCUSSION

Although in the past the stroma was considered as a support tissue for neoplastic cells, the results obtained in numerous scientific investigations indicate that it can regulate the processes of tumor invasion and metastasis^2^^,^^4^^,^^5^. Despite the diversity of cell types in the tumor stroma - blood and lymphatic endothelial cells, inflammatory cells, immune system cells - studied have suggested that myofibroblasts are among the main cell types involved in the promotion of malignant phenotypes^1^, ^2^, ^3^, ^4^, ^5^^,^^10^.

Although few in number, the studies done on oral SCC suggest myofibroblasts have an important role in the process of tumor invasion, in addition to correlating with local disease recurrence and reduced patient survival^3^^,^^5^^,^^6^^,^^33^, ^34^, ^35^, ^36^. The molecular mechanism by which myofibroblasts impact the biological behavior of oral SCC is yet to be fully understood, but it may involve the modulation of the expression of various growth factors, cytokines, ECM components, and proteolytic enzymes, particularly matrix metalloproteinases (MMPs), as reported in studies concerning other malignant neoplasms^1^^,^^5^^,^^8^.

Given their ability to synthesize and secrete different growth factors such as HGF, PDGF, KGF, and granulocyte-macrophage colony-stimulating factor (GM-CSF)^1^^,^^2^^,^^38^, research has indicated that myofibroblasts play an important role in neoplastic cell proliferation^10^^,^^39^. Despite this statement, Lewis et al.^6^ observed that oral cancer cells cultured in a fibroblast conditioned medium had similar cell proliferation rates to specimens cultured in myofibroblast conditioned medium. By their turn, Kellermann et al.^8^ reported significant increases in cell proliferation rates in oral cancer cells cultured in myofibroblast conditioned medium. The effect myofibroblasts appear to have on oral cancer cell proliferation rates was further supported by Sobral et al.^5^. Additionally, these authors identified that the effects myofibroblasts exert upon oral cancer cell proliferation rates arise from the secretion of activin A, a protein from the TGF-β family.

Research has indicated that myofibroblasts may foster the migration of neoplastic cells^17^^,^^40^, probably by secreting proteases that degrade components of the extracellular matrix such as MMPs −2, −3 and −9^4^^,^^41^, urokinase-type plasminogen activator^2^^,^^39^, and fibroblast activation protein^40^. Along the same lines, Sobral et al.^5^ found, on an *in vitro* study, that myofibroblasts triggered the invasion of oral cancer cells in a Matrigel matrix, an event accompanied by increased metalloproteinase synthesis in tumor cells, particularly MMPs −1, −2, −9, and −13. Additionally, the authors observed a significant *in vivo* correlation through zymography between the presence of myofibroblasts and enzymatic activity of MMPs −2 and −9 in samples of oral cancer cells. Other studies regarding oral SCC have yet revealed that myofibroblasts promote tumor invasion by secreting chemokines^42^ and synthesizing specific ECM components such as some laminin isoforms^43^.

Despite the various mechanisms presented previously and the research suggesting an important role for myofibroblasts in oral SCC invasion and correlating the presence of myofibroblasts to local disease recurrence and diminished patient survival^3^^,^^5^^,^^6^^,^^33^, ^34^, ^35^, ^36^, immunohistochemistry findings revealed that significant percentages of oral SCC cases have low myofibroblast counts or are negative for this cell type.

Kellermann et al.^8^ reported 39.48% (n = 15) of oral SCC cases categorized as negative for stromal myofibroblasts. Additionally, 11 (47.82%) of the 23 oral SCC patients positive for myofibroblasts had low counts of this cell type. Nonetheless, the authors observed a significant correlation between high myofibroblast counts in the tumor stroma, regional node involvement, advanced clinical staging, and regional (nodal) recurrence. On another study, De-Assis et al.^9^ described high myofibroblast counts in 15 (36.58%) of 41 oral SCC patients tested through immunohistochemistry, most of whom had high histological grades of malignant disease^9^. Altogether, the findings in these studies showed that myofibroblasts were not present in all cases of oral SCC, in addition to suggesting that stromal myofibroblast count could aid in the identification of biologically more aggressive neoplasms.

In addition to not being abundantly present in all cases of oral SCC, several studies have indicated that myofibroblasts were involved only in later-stage oral cancers. Leukoplakic lesions with varying degrees of epithelial dysplasia did not present myofibroblasts in the lamina propria, as the appearance of significant amounts of myofibroblasts was associated with invasion of the underlying connective tissue^3^^,^^9^^,^^13^. According to Etemad-Moghadam et al.^13^, the appearance of myofibroblasts in oral SCC may be related to the effects carcinomatous epithelium exerts upon the neighboring stroma.

## CONCLUSION

Myofibroblasts are a relevant component in the stroma of oral cancer cells, although they are not present in all oral SCC cases. Although few in number, *in vitro* studies indicate that myofibroblasts are involved in the contractile function in the invasion process of oral SCC, probably as a result of the modulation of the expression of growth factors, cytokines, ECM components, and proteolytic enzymes. Additionally, clinical, pathology, and immunohistochemistry tests have correlated the presence of high myofibroblast counts in oral cancer cell stroma with local disease recurrence and reduced patient survival. Despite the importance of these findings, the limited number of studies on the topic calls for additional research so that the molecular mechanisms by which myofibroblasts impact the biological behavior of oral SCC are further clarified.
